# Expression of semaphorin 3A (SEMA3A) in breast cancer subtypes

**DOI:** 10.1038/s41598-024-51796-z

**Published:** 2024-01-23

**Authors:** Natalia Andryszak, Paweł Kurzawa, Monika Krzyżaniak, Marek Ruchała, Michał Nowicki, Dariusz Iżycki, Rafał Czepczyński

**Affiliations:** 1 Department of Endocrinology, Metabolism and Internal Medicine, Poznan University of Medical Sciences, Poznan, Poland; 2 Department of Oncological Pathology, University Clinical Hospital in Poznan, Poznan University of Medical Sciences, Poznan, Poland; 3 Department of Histology and Embryology, Poznan University of Medical Sciences, 60-781 Poznan, Poland; 4 Department of Cancer Immunology, Poznan University of Medical Sciences, Poznan, Poland

**Keywords:** Breast cancer, Tumour biomarkers

## Abstract

Breast cancer is a major health concern, and its accurate diagnosis and management depend on identifying its histological type and biological subtype. Semaphorin-3A (SEMA3A) is a membrane protein with diverse roles in cellular processes, including cancer progression and angiogenesis regulation. However, its role in breast cancer remains poorly understood. This study aimed to evaluate SEMA3A expression in breast cancer and investigate its distribution across breast cancer subtypes: luminal A, luminal B, HER2-positive, and triple-negative breast cancer (TNBC). Immunohistochemical evaluation was performed on 98 breast cancer patients' tumor specimens, and SEMA3A expression was assessed in tumor cells and vessels. The study included the analysis of the Ki67 proliferation index, estrogen receptor (ER) expression, progesterone receptor (PR) expression, and HER2 status in conjunction with SEMA3A expression. Analysis indicated positive expression of SEMA3A in breast cancer cells in 60 out of 98 cases. SEMA3A expression correlated positively with Ki67 levels in tumor cells (p = 0.0005, R Spearman 0.338). Notably, a negative correlation was found between SEMA3A expression and ER and PR levels in tumor cells (p = 0.04, Spearman's R = − 0.21 and p = 0.016, Spearman's R = − 0.25 respectively). HER2 status did not significantly influence SEMA3A expression. The study demonstrated positive SEMA3A expression in tumor vessels across all subtypes in 91 out of 98 cases, suggesting its involvement in endothelial cell function. However, no significant differences in SEMA3A expression were observed between breast cancer subtypes either in vessels or tumor cells. These findings suggest that elevated SEMA3A expression may be associated with worse prognosis in breast cancer, especially in ER- and PR-negative tumors. Further investigations are warranted to fully comprehend the role of SEMA3A in breast cancer biology, which may lead to the identification of novel therapeutic targets and personalized treatment strategies for breast cancer patients.

## Introduction

Breast cancer is the most common cancer in women and the second leading cause of cancer-related deaths after lung cancer^1^, as reported by the American Cancer Society. The accurate diagnosis and management of breast cancer depend on identifying the histological type of the tumor and determining its biological subtype by the means of immunohistochemical evaluation. This classification is based on the assessment of steroid receptor expression, such as estrogen and progesterone receptors, HER2 status, and the Ki-67 proliferation index. These factors categorize breast cancer into main biological subtypes: luminal A, luminal B, HER2-positive (within HER2-luminal and nonluminal), and triple-negative breast cancer (TNBC). This categorization plays a crucial role in guiding appropriate systemic treatment strategies^2^.

Semaphorin-3A (SEMA3A) is a significant membrane protein belonging to the semaphorin family, which plays diverse roles in cellular processes. Initially recognized for its involvement in axonal guidance during embryonic neural development, SEMA3A has recently gained attention for its expanded functions and vital roles in neogenesis and malignant pathology^3^. Notably, it is expressed on endothelial cells and interacts with neuropilins (NRP) and vascular endothelial growth factor (VEGF), suggesting its regulatory influence on angiogenesis and cancer progression. In addition, VEGF/NRP interactions are proangiogenic, while SEMA3/NRP interactions are inhibitory^4^. However, SEMA3A expression varies across cancer subtypes and can either be correlated with a better prognosis, by inhibiting tumor growth and angiogenesis, or induce cancer cell invasiveness and cancer progression. In various cancers, such as ovarian, gastric or non-small cell lung cancer, SEMA3A has been found to be downregulated, and this decreased expression has been associated with worse prognoses, increased malignancy, deeper tumor invasion, enhanced metastasis formation, and altered cell adhesion and migration^1,5–8^. On the contrary, in pancreatic cancer and prostate cancer, SEMA3A overexpression has been found and it could be linked to metastatic progression^9,10^. Similarly, in hepatocellular carcinoma SEMA3A promoted tumor proliferation and migration^11^. Additionally, studies suggest that SEMA3A overexpression and its NRP1 binding may serve as potential targets for the treatment of human pancreatic cancer^12^. Also in glioblastoma, a tumor inhibitory effect of anti-SEMA3A IgG antibody was found by Jaehyun et al. demonstrating thereby its potential relevance as a therapeutic agent^13^.

Regarding breast cancer, there are different studies exploring various influences of SEMA3A^14^. For example, Mishra et al. reported on a mechanism by which SEMA3A attenuates tumor growth and angiogenesis by inducing the expression of the tumor suppressor gene MelCAM in a breast cancer model^15^. Casazza et al. demonstrated that SEMA3A overexpression inhibits vessel formation and increases tumor hypoxia and necrosis in an in vivo mice model^16^. Conversely, Gehler et al. showed that SEMA3A increases cancer cell migration and spreading^17^, while Wei-Wei et al. indicated increased SEMA3A expression in breast cancer bone metastases cells^18^. Further studies are warranted to comprehensively evaluate the role of SEMA3A in breast cancer. While existing research has shed some light on SEMA3A's involvement in various cancers, including breast cancer, a more in-depth investigation is necessary to fully understand its implications in this specific context and its possible clinical impact.

The objective of our study was to evaluate SEMA3A expression in breast cancer and to investigate its distribution across breast cancer subtypes. Additionally, we sought to explore how immunohistochemical subtypes of breast cancer might impact SEMA3A expression.

## Methods

**Figure 1:**
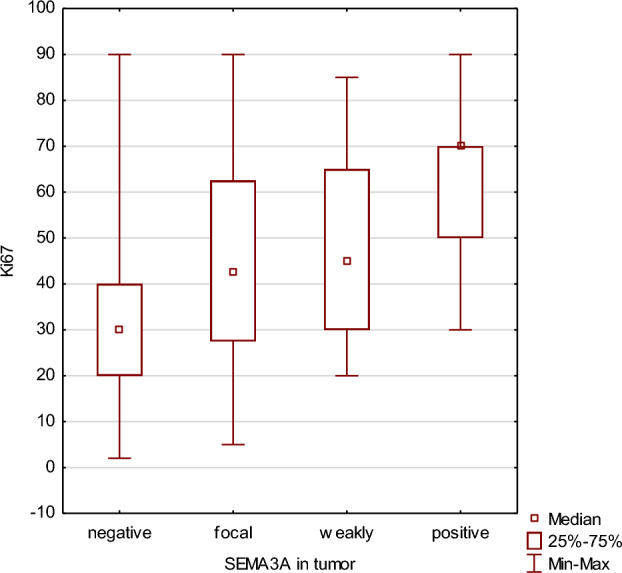
Correlation between Ki67 level and SEMA3A expression in tumor cells.

This retrospective study received approval from the Institutional Bioethical Committee. The study was based on the immunohistochemical evaluation of SEMA3A expression in the breast cancer specimens obtained surgically from the primary tumor. Consecutive patients with breast cancer treated in a tertiary cancer center were considered for the inclusion to the study. In order to obtain a more homogenous population, only female patients with stage I or II breast cancer aged less than 50 years were included. Exclusion criteria comprised a history of another malignancy or autoimmune disease as well as incomplete data. Immunohistochemistry data, including the expression of estrogen and progesterone receptors, HER2 status, and Ki67 level, were analyzed in the tumor samples (Fig.1).

The classification of breast cancer subtypes included into our study:

- Luminal A: ER positive and/or PR positive, absence of HER2 and low expression of cell proliferation marker Ki-67 (less than 20%)
- Luminal B: ER positive and/or PR positive, absence of HER2 and a high expression of Ki67 (greater than 20%).
- HER2-positive with two distinguished subgroups:
  - HER2-luminal (E+, PR+, HER2+ and Ki-67:15–30%)
  - HER2-nonluminal (HER2+, E−, PR−)
- Triple-negative: ER-negative, PR-negative, and HER2-negative

The study involved the analysis of data from 2039 consecutive patients with breast cancer who were hospitalized in the Oncology Department between the years 2017 and 2022. Among them, 500 patients had complete data and good quality paraffin sections available for analysis. After applying the predefined inclusion criteria, a total of 98 patients were retrieved and included in the study. The age range of the participants was between 32 and 50 years, with a mean age of 41.1 years.

The analysis focused on formalin-fixed, paraffin-embedded tissue blocks obtained from core biopsies specimen collected from each of the patient before treatment, specifically at the time of diagnosis. Detailed clinicopathological data were collected from pathology and clinical records.

### Immunohistochemistry

For expression of SEMA3A protein immunohistochemical analysis was performed on glass slides. They contained tissue samples from 98 patients. Each slide contained specimen from core biopsy of 1 patient. Apart from that each slide contained external control with 4 different normal tissue cores: tonsil, appendix, liver, and pancreas.

Serial 4-µm tissue cut sections obtained from the paraffin block containing core biopsy of breast cancer was applied to special immunohistochemistry coated slides with external control cores cut before. Then, slides were baked for at 60 min in 60 °C.

A Semaphorin 3A, clone EPR19367 was used to demonstrate expression of the secreted protein. Deparaffinization, rehydration and heat-induced epitope retrieval (HIER) was performed using 3-in-1 procedure in PT Link Module (Agilent Technologies, Denmark) by processing sections in a citrate buffer (pH = 6.1) for 20 min at a temperature of 97 °C. After cooling and washing in EnVision FLEX Wash Buffer, endogenous peroxidase was blocked for 10 min at room temperature. Incubation of the primary antibody SEMA3A (Abcam) took 30 min. Labelled polymer—HRP was incubated for 20 min. EnVision FLEX (K8002) was used as detection system. Staining was performed on a fully automated immunohistochemistry slide stainer Autostainer Link 48 (Agilent Technologies, Denmark).

The antigen was localized using Chromogen DAB-3.3 applied on all tissue slices. Slides were then stained with Hematoxylin (K8008) for 5 min. After staining, the sections were dehydrated, cleared and mounted using permanent mounting method.

The SEMA3A expression was evaluated using four categories: negative, focal, weakly positive, and strongly positive. The expression was evaluated on both, tumor cells and tumor vessels. Positive expression was reported if it occurred in the cell membrane or cytoplasm or both. The evaluation of SEMA3a reactivity in tumor cells and tumor vessels was conducted in a blinded manner, without knowledge of the clinical data.

Each slide contained specimen from 1 patient and external control which was used in pathology department for a daily quality control purpose. The external control contained 4 different normal tissue cores: tonsil, appendix, liver and pancreas. Daily quality control was used to exclude any false-positive staining reactions and verify the used detection system for “nonspecific” binding of the components.

**Figure 2:**
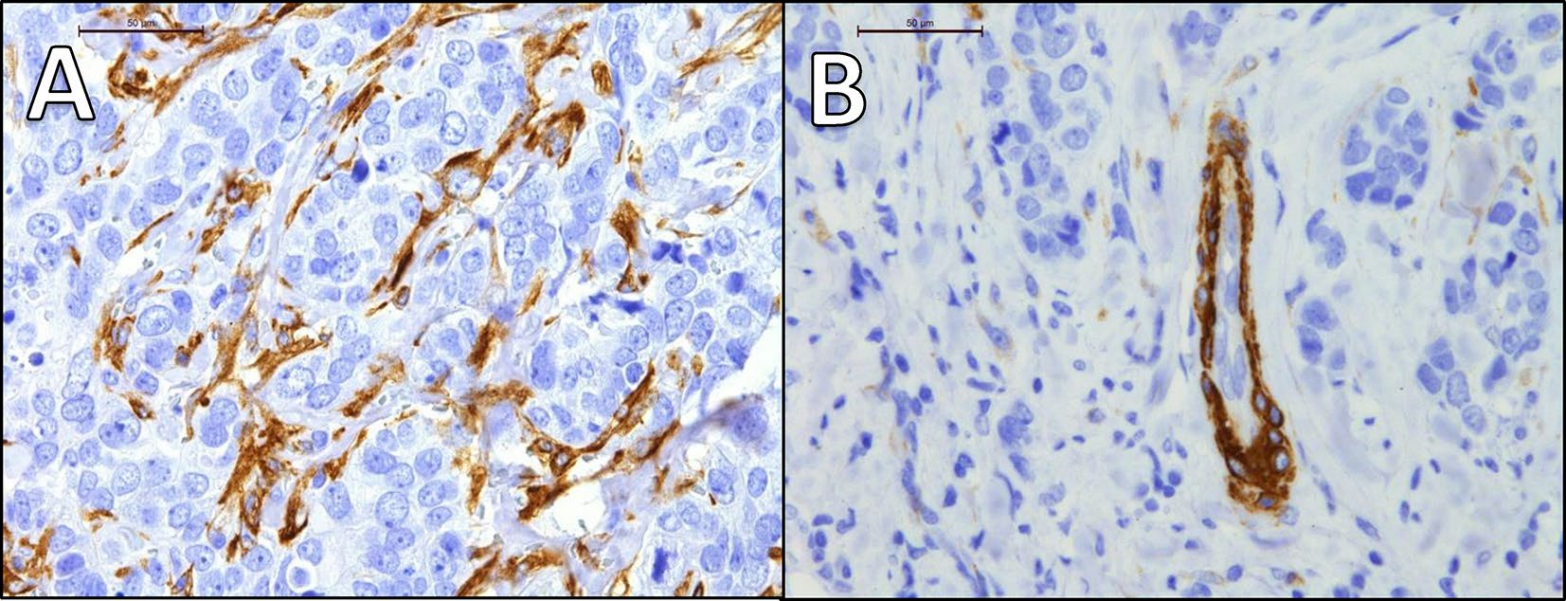
(**A**) and (**B**) presents case of one patient with triple negative breast cancer (TNBC), Ki67 90%, grade 3. Immunohistochemistry revealed strongly positive expression of SEMA3A in tumor cells (cytoplasm + membrane) (**A**) and positive expression of SEMA3A in tumor-associated vessels (**B**). The patient was diagnosed at stage IIA. (**A**) mag. ×400, (**B**) mag. ×400.

**Figure 3:**
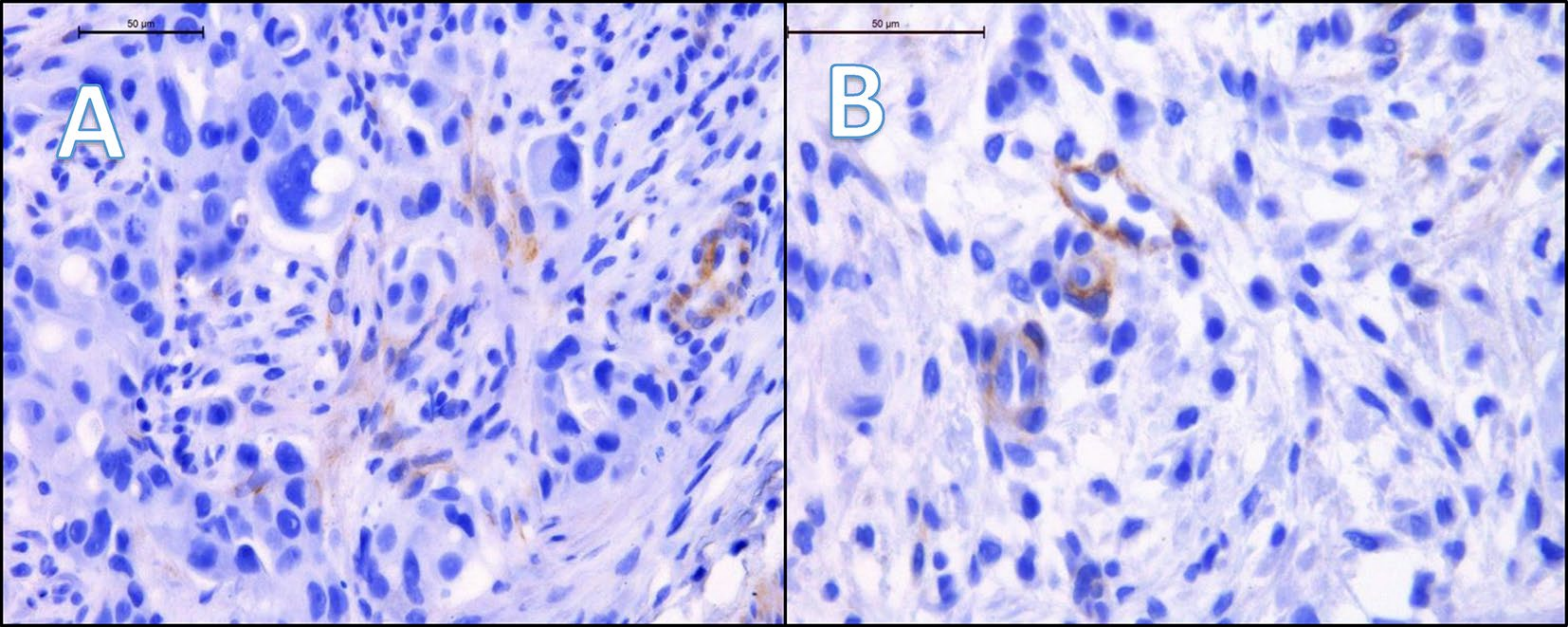
(**A**) and (**B**) presents case of one patient with HER2 non-luminal, Ki67 50%, grade 2. Immunohistochemistry revealed weakly positive expression of SEMA3A in tumor cells (cytoplasm + membrane) (**A**) and weakly positive expression of SEMA3A in tumor-associated vessels (**B**). The patient was diagnosed at stage IIA. (**A**) mag. 40, (**B**) mag. 63. (**A**) mag. ×400, (**B**) mag. ×630.

**Figure 4:**
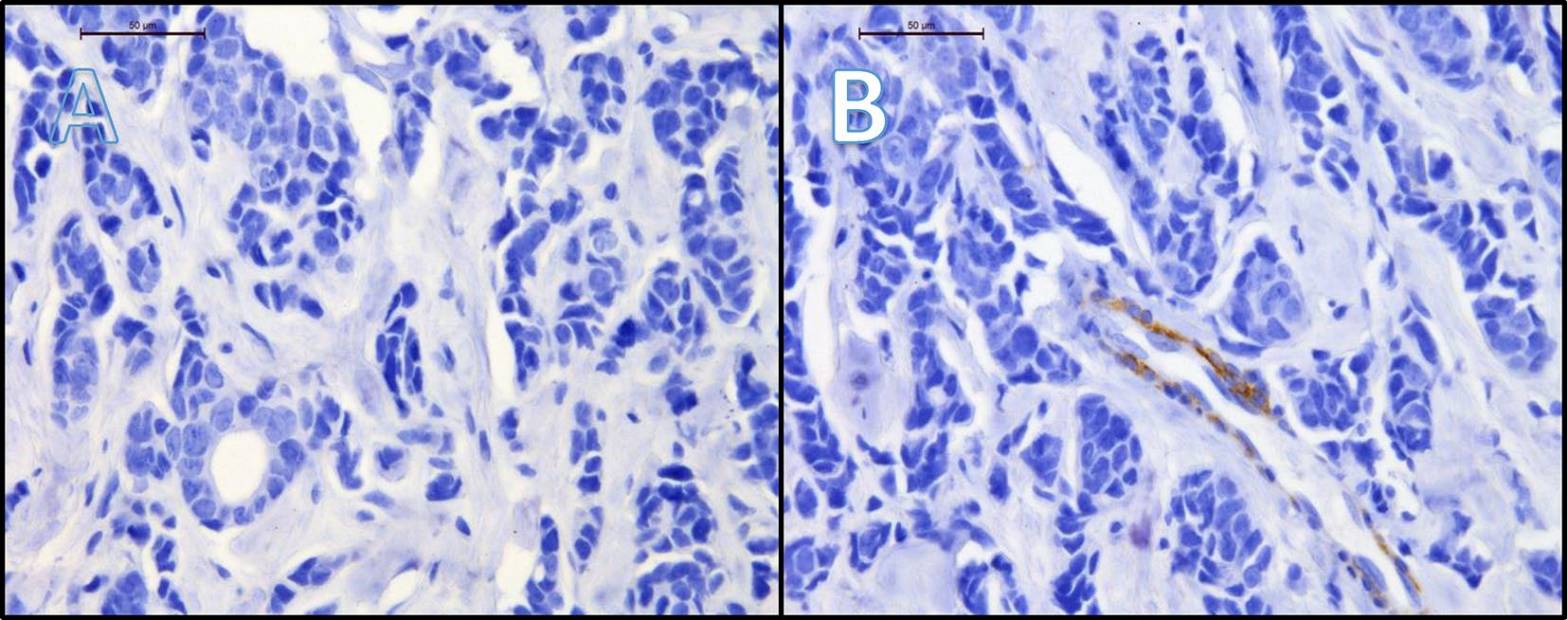
(**A**) and (**B**) presents case of one patient with luminal A breast cancer, 90% estrogen receptor expression, 70% progesterone receptor expression, Ki67 2%, grade 2. Immunohistochemistry revealed negative expression of SEMA3A in tumor cells (cytoplasm + membrane) (**A**) and weakly positive expression of SEMA3A in tumor-associated vessels (**B**). The patient was diagnosed at stage IIA. (**A**) mag. 40, (**B**) mag. 40. (**A**) mag. ×400, (**B**) mag. ×400.

Microscopic slide images (Figs. 2, 3, 4) were taken using Leica microsope and Leica camera with 20×, 40× and 63× objectives.

### Statistical analysis

The calculations were performed using the Statistica 13 software by TIBCO and PQStat software by PQStat Software. A significance level of α = 0.05 was adopted. A result was considered statistically significant when p < α. The normality of variable distributions was assessed using the Shapiro–Wilk test. Due to non-normal distribution, the Kruskal–Wallis test with the Dunna–Bonferroni multiple comparison test was used to compare variables among multiple groups. The Jonckheere–Terpstra test was used to determine if there was a significant trend. The Spearman's rank correlation coefficient (Rs) was calculated to examine the relationships between variables. The Fisher–Freeman–Halton test was used to analyze the associations between categorical variables.

### Ethics approval and consent to participate

This study was conducted in accordance with the principles outlined in the Declaration of Helsinki. The Institutional Review Board of Poznan University of Medical Sciences determined that this retrospective study did not involve research experimentation. Informed consent has been obtained from all patients to collect specimen for tissue biopsy.

## Results

98 breast cancer patients were included in the study, with mean age of 41.1 ± 8.2 years, with the following distribution among the subtypes:

- HER2-positive: 29 cases, including 12 HER2 non-luminal and 17 HER2 luminal cases.
- Triple-negative breast cancer (TNBC): 19 cases.
- Luminal A: 25 cases.
- Luminal B: 25 cases.

The expression of SEMA3A in the tumor cells was negative in 38 cases and positive in 60 cases: 5 cases strongly positive, 17-weakly positive and 36—focal. SEMA3A expression in tumor vessels was found in 91 out of 98 cases; in 20 cases the expression was strongly positive, in 37—weakly positive and in 34—focal expression was present.

We analyzed differences of SEMA3A expression between cancer subtypes. No significant difference in SEMA3A expression was observed in the tumor cells between subtypes (p = 0.1419) (Table 1) and in tumor vessels among different breast cancer subtypes (luminal A/B, HER2 and TNBC) (p = 0.2263) (Table 2).

**Table 1:** Distribution of SEMA3A expression level in tumor cells between subtypes.

Expression in tumor	Negative	Focal	Weak	Strong	All
TNBC	5	5	6	3	19
HER luminal	9	6	2	0	17
HER2 nonluminal	3	3	5	1	12
Luminal A	13	9	3	0	25
Luminal B	9	13	2	1	25
ALL	39	36	18	5	98

**Table 2:** Distribution of SEMA3A expression level in tumor vessels between subtypes.

Expression in vessels	Negative	Focal	Weak	Strong	All
TNBC	1	7	5	6	19
HER luminal	0	9	6	2	17
HER2 nonluminal	1	3	3	5	12
Luminal A	1	4	16	4	25
Luminal B	3	8	8	6	25
ALL	6	31	38	23	98

We analyzed correlations between level of SEMA3A expression and various immunohistochemical factors of breast cancer subtypes (Tables 3 and 4).

**Table 3:** SEM3A expression in tumor cells in relation to receptor and Ki67 status.

	N	Mean	Median	Minimum	Maximum	SD
SEMA3A negative expression in tumor cells						
ER%	38	61.84	80.00	0.00	95.00	37.71
PR%	38	39.00	40.00	0.00	95.00	37.30
Ki67	38	34.03	30.00	2.00	90.00	21.50
SEMA3A focal expression in tumor cells						
ER%	36	56.39	75.00	0.00	95.00	39.09
PR%	36	35.56	20.00	0.00	95.00	37.24
Ki67	36	45.61	42.50	5.00	90.00	23.07
SEMA3A weak expression in tumor cells						
ER%	17	37.94	0.00	0.00	95.00	46.77
PR%	17	19.35	0.00	0.00	95.00	35.50
Ki67	17	50.29	45.00	20.00	85.00	22.11
SEMA3A strong expression in tumor cells						
ER%	7	5.00	0.00	0.00	25.00	11.18
PR%	7	0.00	0.00	0.00	0.00	0.00
Ki67	7	62.00	70.00	30.00	90.00	22.80

*ER* estrogen receptor percentage, *PR* progesterone receptor percentage.

**Table 4:** SEM3A expression in tumor vessels in relation to receptor and Ki67 status.

	N	Mean	Median	Minimum	Maximum	SD
SEMA3A negative expression in tumor vessels						
ER%	7	55.00	75.00	0.00	90.00	38.41
PR%	7	37.00	25.00	0.00	80.00	40.56
Ki67	7	33.75	32.00	20.00	50.00	12.50
SEMA3A focal expression in tumor vessels						
ER%	34	44.12	55.00	0.00	95.00	41.22
PR%	34	24.68	0.50	0.00	95.00	35.02
Ki67	34	42.94	35.00	15.00	90.00	21.64
SEMA3A weak expression in tumor vessels						
ER%	37	66.08	90.00	0.00	95.00	38.73
PR%	37	44.19	40.00	0.00	95.00	37.50
Ki67	37	42.08	40.00	2.00	90.00	23.61
SEMA3A strong expression in tumor vessels						
ER%	20	41.50	27.50	0.00	95.00	41.49
PR%	20	21.60	0.00	0.00	90.00	34.81
Ki67	20	45.70	45.00	2.00	90.00	27.73

*ER* estrogen receptor percentage, *PR* progesterone receptor percentage.

### Ki67 level

There was no significant correlation between SEMA3A expression and Ki67 level in tumor vessels (p = 0.89). However, a correlation between SEMA3A expression and Ki67 level was found in the tumor cells (p = 0.0005, R Spearman 0.338) (Fig. 1).

### Estrogen receptor expression

Tumors with estrogen receptor expression exhibited significantly lower levels of SEMA3A expression in the tumor cells (p = 0.0024). However, there were no significant differences in estrogen receptor expression within the tumor vessels (p = 0.011). A significant negative correlation was observed between the percentage of estrogen receptor expression and SEMA3A expression in the tumor (p = 0.04, Spearman's R = − 0.21), but not in the vessels (p = 0.57).

### Progesterone receptor expression

Tumors with progesterone receptor expression exhibited significantly lower levels of SEMA3A expression in the tumor cells (p = 0.0097). However, there were no significant differences in progesterone receptor expression within the tumor vessels (p = 0.11). A significant negative correlation was observed between the percentage of progesterone receptor expression and SEMA3A expression in the tumor (p = 0.016, Spearman's R = − 0.25), but not in the vessels (p = 0.82).

### HER2 status

HER2 status did not significantly influence SEMA3A expression, either in the tumor (p = 0.83) or in the vessels (p = 0.09).

## Discussion

The semaphorin family comprises a diverse group of proteins known to play critical roles in various biological processes, including neuronal development, immune response, and cancer pathophysiology. These proteins are categorized into eight classes based on their structural elements and distribution. Some members of the semaphorin classes have been identified as having antiangiogenic and antimetastatic properties, while others act as proangiogenic and prometastatic factors. Only class 3 semaphorins are secreted membrane proteins and in our paper, our primary focus is on protein SEMA3A^3^.

Neuropilins (NRPs) are receptors for the class 3 semaphorins, including SEMA3A, which are known for their role in guiding axons during development and their binding with the vascular endothelial growth factor (VEGF). While the initial studies on SEMA3s and NRPs highlighted their function in axon guidance, it has become increasingly evident that these proteins also play significant roles in vascular and tumor biology^3,4^. Gastric, ovarian, or lung cancer, exhibit a negative correlation between SEMA3A expression and cancer progression. However, there are certain cancers, like prostate cancer, glioblastoma, or pancreatic cancer, where overexpression of SEMA3A is associated with a poor prognosis and the development of metastases.

The results of our study provided evidence of positive expression of SEMA3A in breast cancer (Fig. 2). We observed a significant positive correlation between SEMA3A expression and Ki67 levels in both tumor cells and tumor vessels. This finding suggests that as SEMA3A levels increase, there is a concurrent increase in cell proliferation, which is associated with a worse prognosis. Surprisingly, we did not find significant differences in SEMA3A expression between different breast cancer subtypes. However, we did observe a significant negative correlation between SEMA3A expression and both estrogen and progesterone receptor levels. Luminal breast cancer subtypes, characterized by positive steroid receptor expression, are known to have a better prognosis and lower proliferation rates. Therefore, the negative correlation between SEMA3A and steroid receptor levels indicates that elevated SEMA3A expression may have a detrimental impact on prognosis in breast cancer.

In our study, we conducted an analysis of SEMA3A expression in both, tumor cells and vessels. Interestingly, the vascular expression of SEMA3A was observed in a great majority of cases, with only six out of 98 patients showing no expression. Additionally, we did not find any significant differences in SEMA3A expression between different breast cancer subtypes in vessels, suggesting that the vascular expression may be related to physiological role of SEMA3A in endothelial cells (Fig. 3). Interestingly, Saton et al. conducted a histological analysis, which revealed that there is a SEMA3A expression in normal breast tissue and it decreased with the transition from in situ to invasive cancer^19^. However, they observed an increased expression of neurolipin-1, a SEMA3A binding protein related to angiogenesis, in breast cancer cells as compared to normal breast tissue. The most significant differences in SEMA3A expression were observed within the tumor cells themselves, indicating its potential role in tumor proliferation and cancer progression. Additionally, Mamoor et al. demonstrated significantly higher expression of SEMA3A in primary breast cancer tumors compared to breast cancer brain metastasis, suggesting that SEMA3A expression could potentially influence the metastatic cascade and contribute to the complex mechanisms of breast cancer metastasis. Therefore, SEMA3A expression might have implications for disease prognosis and treatment strategies.^20^. They suggested that SEMA3A expression could potentially influence the metastatic cascade and contribute to the complex mechanisms of breast cancer metastasis. Given its impact on tumor cell behavior, SEMA3A expression may have implications for disease prognosis and treatment strategies.

Our primary focus was the early-stage breast cancer, as there has been relatively less exploration of SEMA3A in this context, as compared to metastatic research. Patients younger than 50 years old were included into the study to obtain more homogenous subgroups and to exclude comorbidities, which may affect SEMA3A expression.

Fei et al. conducted a study confirming that SEMA3A expression was upregulated in patients with castration-resistant prostate cancer, and its increased expression was negatively correlated with the prognosis. Moreover, they proposed that targeting SEMA3A and its receptor NRP1 could hold potential as a therapeutic approach for patients with androgen-resistant prostate cancer^10^. Jaehyun et al. demonstrated tumor inhibitory effect of anti-SEMA3A IgG antibody in glioblastoma progression and presented its potential relevance as a therapeutic agent^12^. The proven strongly positive expression of SEMA3A in breast cancer tumor cells, its involvement in vascular development and tumor biology suggests potential importance of SEMA3A as therapeutic target in breast cancer treatment.

## Conclusions

In conclusion, our study provides valuable insights into the complex role of SEMA3A in breast cancer biology. Understanding its implications for different breast cancer subtypes and its impact on prognosis may pave the way for the identification of novel therapeutic targets and the development of personalized treatment strategies for breast cancer patients. However, further research is necessary to unravel the underlying mechanisms and validate these findings in larger patient cohorts.

## Data Availability

The datasets used and/or analysed during the current study available from the corresponding author on reasonable request.
